# The NMDA receptor GluN2C subunit controls cortical excitatory-inhibitory balance, neuronal oscillations and cognitive function

**DOI:** 10.1038/srep38321

**Published:** 2016-12-06

**Authors:** Subhash C. Gupta, Aparna Ravikrishnan, Jinxu Liu, Zhihao Mao, Ratnamala Pavuluri, Brandon G. Hillman, Pauravi J. Gandhi, Dustin J. Stairs, Ming Li, Rajesh R. Ugale, Daniel T. Monaghan, Shashank M. Dravid

**Affiliations:** 1 Department of Pharmacology, Omaha, NE 68178, USA; 2 Department of Pharmacology and Experimental Therapeutics, University of Nebraska-Medical Center, Omaha, NE, 68198, USA; 3 Psychology, Creighton University, Omaha, NE 68178, USA; 4 Department of Psychology, University of Nebraska-Lincoln, Lincoln, NE 68588, USA; 5 Department of Pharmaceutical Sciences, R.T.M. Nagpur University, Nagpur, Maharashtra 440033, India

## Abstract

Despite strong evidence for NMDA receptor (NMDAR) hypofunction as an underlying factor for cognitive disorders, the precise roles of various NMDAR subtypes remains unknown. The GluN2C-containing NMDARs exhibit unique biophysical properties and expression pattern, and lower expression of GluN2C subunit has been reported in postmortem brains from schizophrenia patients. We found that loss of GluN2C subunit leads to a shift in cortical excitatory-inhibitory balance towards greater inhibition. Specifically, pyramidal neurons in the medial prefrontal cortex (mPFC) of GluN2C knockout mice have reduced mEPSC frequency and dendritic spine density and a contrasting higher frequency of mIPSCs. In addition a greater number of perisomatic GAD67 puncta was observed suggesting a potential increase in parvalbumin interneuron inputs. At a network level the GluN2C knockout mice were found to have a more robust increase in power of oscillations in response to NMDAR blocker MK-801. Furthermore, GluN2C heterozygous and knockout mice exhibited abnormalities in cognition and sensorimotor gating. Our results demonstrate that loss of GluN2C subunit leads to cortical excitatory-inhibitory imbalance and abnormal neuronal oscillations associated with neurodevelopmental disorders.

Glutamate is the major excitatory neurotransmitter in the mammalian central nervous system. There are four classes of ionotropic glutamate receptors (iGluRs) classified on the basis of sequence similarity and pharmacology. One class of the iGluRs is the *N-*methyl-*D*-aspartate receptor (NMDAR), which has been shown to play a key role in synaptic plasticity and neural development. NMDARs are tetrameric receptors composed of two obligatory GluN1 subunits and generally two GluN2 subunits. There are four GluN2 subtypes (GluN2A-D) which confer different biophysical, signaling and pharmacological properties to the receptor. Dysregulation of NMDAR function and signaling has been proposed as an underlying pathophysiology in neurodevelopmental disorders. This hypothesis is particularly strong for schizophrenia since administration of NMDAR channel blockers in humans replicates the symptoms in schizophrenia^1^,^2^,^3^ and agonists for the GluN1 subunit glycine, D-serine and D-cycloserine partly alleviate schizophrenia symptoms^4^,^5^. Despite these converging findings, our understanding of the subtype selective roles of NMDARs which may allow selective targeting of neural circuits underlying mental disorders is lacking.

The GluN2C subunit is enriched in the cerebellar granule cells, but knockout of GluN2C subunits does not result in any motor deficits^6^,^7^, consistent with suggestions that GluN2C is redundant with GluN2A in cerebellar function. GluN2C is also enriched in interneurons in the prefrontal cortex^8^,^9^ and mediodorsal thalamus (MDT) relay neurons^8^,^10^ which send axonal inputs to prefrontal cortex (PFC). Previous studies have demonstrated that NMDAR functioning in cortical interneurons, especially PV-positive interneurons, is important for normal neuronal oscillations and cognition^11^,^12^,^13^ and inputs from MDT regulate cortical function^14^. Furthermore, GluN2C expression in interneurons in reticular nuclei of the thalamus (nRT) appears to modulate delta oscillations in the telencephalon^15^ which can then modulate gamma oscillations in cortex^16^. Thus, the site of GluN2C expression together with its unique biophysical properties (lower Mg^2+^-block, lack of desensitization and high agonist affinity)^17^, suggest that GluN2C-containing receptors may have significant impact on cortical function. Interestingly, independent groups have found lower GluN2C subunit expression in the PFC and thalamus in post-mortem brains from schizophrenic patients^18^,^19^,^20^,^21^,^22^. Using the GluN2C genetic knockout model we tested the hypothesis that GluN2C ablation leads to cortical dysfunction associated with neurodevelopmental disorders. The results presented herein demonstrate that reduction in GluN2C-containing NMDARs leads to cortical excitatory-inhibitory imbalance and abnormal neuronal oscillations and behavioral phenotypes reminiscent of neurodevelopmental disorders.

## Results

### Ablation of GluN2C subunits leads to imbalance between excitatory and inhibitory neurotransmission in local cortical circuitry

**Figure 1:**
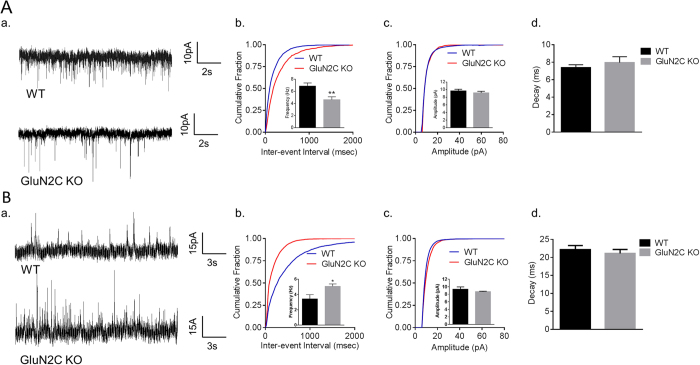
Opposing alterations in excitatory and inhibitory activity of layer V pyramidal neurons in the mPFC of GluN2C KO mice. (**A**) A significant reduction was observed in the frequency, but not amplitude or decay, of mEPSCs of layer V pyramidal neurons in the mPFC of GluN2C KO mice. Representative traces (a); Quantitative data (n = 20 for WT and 13 for KO from 4–5 mice/genotype) cumulative probability plots of inter-event interval and frequency in inset, significant reduction in mEPSC frequency in GluN2C KO was observed (unpaired t-test with Welch’s correction, **P = 0.0035) (b); cumulative probability plot for amplitude and mean amplitude in inset (c) and decay (d) of mEPSCs. (**B**) A significant increase was observed in the frequency, but not amplitude or decay of mIPSCs from layer V pyramidal neurons in the mPFC of GluN2C KO mice. Representative traces (a); Quantitative data (n = 11 for WT and 16 for KO from 4–5 mice/genotype) cumulative probability plots of inter-event interval and frequency in the inset, significant increase in frequency of mIPSC in GluN2 KO was observed (unpaired t-test with Welch’s correction, *P = 0.0204); cumulative plot for amplitude and mean amplitude in inset (c); and decay (d) of mIPSCs.

The dorsolateral prefrontal cortex (DLPFC) is a critical site for controlling behavioral flexibility and social behavior, and dysfunction of the DLPFC is implicated in schizophrenia and other cognitive disorders. Consistent with this interpretation, several cellular abnormalities have been reported in the DLPFC of schizophrenic patients, which represent an imbalance in excitatory and inhibitory neurotransmission (E/I imbalance)^23^,^24^. To this extent, we assessed excitatory and inhibitory neurotransmission in GluN2C KO mice. In electrophysiological recordings, we found that layer V medial prefrontal cortex (mPFC) pyramidal neurons from GluN2C KO mice have a significantly lower frequency of mEPSCs compared to WT controls (Fig. 1A, unpaired t-test with Welch’s correction, P = 0.0035, n = 20 for WT and 13 for KO). No significant difference in amplitude or decay of mEPSCs was observed between the genotypes (Fig. 1A). Surprisingly, further analysis revealed that GluN2C KO mice have a significantly higher frequency of mIPSCs compared to WT mice (Fig. 1B, unpaired t-test with Welch’s correction, P = 0.0204, n = 11 for WT and 16 for KO). No significant difference in amplitude or decay of mIPSCs was observed between the genotypes (Fig. 1B). We also performed whole-cell recordings in the absence of TTX and assessed sEPSCs and sIPSCs. None of the sEPSC parameters were altered in GluN2C KO (Supplementary Figure 1A). Although a trend for an increase in frequency of sIPSC was observed this did not reach significance. A modest reduction in sIPSC amplitude was observed (Supplementary Figure 1B). One possibility is that the changes in the excitatory and inhibitory synapse number/function, as indicated by mEPSC and mIPSC assessment, represent compensatory mechanisms to maintain normal action potential and multivesicular release mediated neurotransmission.

### GluN2C KO mice exhibit reduction in dendritic spine density in the mPFC

**Figure 2:**
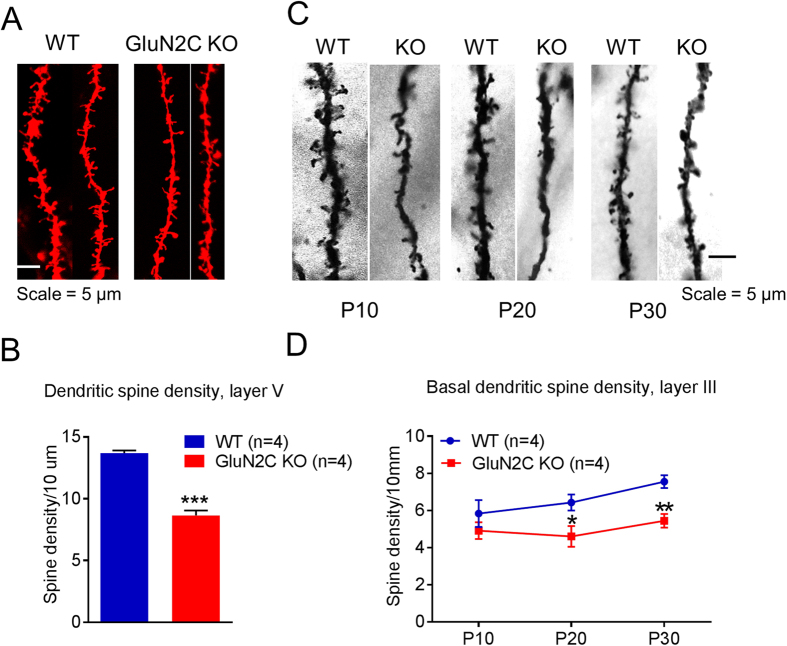
Lower dendritic spine density in medial prefrontal cortex of GluN2C knockout mice. (**A**,**B**) Dendritic spine density of pyramidal neurons in layer V of the medial prefrontal cortex (mPFC) was evaluated using diolistic labeling and Imaris analysis. (**A**) Representative images and (**B**) quantitative results. A significantly lower total spine number was observed in GluN2C KO compared to WT (n = 4 mice/genotype, total of 81 neurons in WT and 76 neurons from GluN2C KO; unpaired t-test, ***P = 0.0002). (**C,D**) Basal dendritic spine density of pyramidal neurons in layer III of the mPFC was determined using Golgi staining. (**C**) Representative images of Golgi-stained sections and (**D**) quantitative results. Basal dendritic spine density was significantly lower in GluN2C KO mice at P30 (n = 4/genotype, unpaired t-test with Welch’s correction, **P = 0.0061 < 0.01) and P20 (n = 4/genotype, unpaired t-test with Welch’s correction, *P = 0.0447), with a trend towards reduction at P10.

To further evaluate deficits in excitatory neurotransmission, we compared the dendritic spine densities in the mPFC of WT and GluN2C KO mice. Using diolistic labeling analysis, we found that the dendritic spine density of layer V pyramidal neurons was significantly lower in the GluN2C KO mice relative to WT controls (Fig. 2A,B, unpaired t-test with Welch’s correction, P = 0.0002, n = 4/genotype). Additional analysis revealed that this reduction was significant for all spine types, including stubby, thin and long, and filopodia (Supplementary Figure 2A,B, unpaired t-test with Welch’s correction, P = 0.01295, stubby; P = 0.0101 thin and long spines and P = 0.0007 for filopodia-like spines), except for mushroom spines. The lack of effect on mushroom spines indicates that loss of GluN2C primarily affects newly formed or less stable synapses. Since synapse formation is an activity-dependent process these findings may be relevant to changes in overall excitatory activity of pyramidal neurons.

A reduction in spine density is a neuropathology associated with schizophrenia^25^. Although, this deficit occurs in layer V of PFC^26^, it is more commonly observed in pyramidal neurons of layer III of the DLPFC^27^,^28^. Given the reports of reduced GluN2C expression in the DLPFC of schizophrenia patients^18^,^21^,^22^ we tested whether loss of GluN2C resemble the neuropathologies observed in schizophrenia patients. To this extent, we studied dendritic morphology in the WT and GluN2C KO mice in layer III neurons of the mPFC. We compared basal and oblique dendritic spine density at 3 ages, which roughly correspond to early childhood (P10) and (P20) and young adulthood (P30) in humans, to determine the age-dependency of the deficit. At P10, we observed a trend towards lower spine density of basal dendrites in GluN2C KO mice (Fig. 2C,D); however, it did not reach significance. A significantly lower spine density was observed in GluN2C KO mice at P20 (Fig. 2C,D, P = 0.0447, unpaired t-test with Welch’s correction, n = 3/genotype) as well as at P30 (Fig. 2C,D, P = 0.0061, unpaired t-test with Welch’s correction, n = 4/genotype). No difference in oblique dendritic spine density was observed at any of the 3 ages between WT and GluN2C KO mice (Supplementary Figure 2B). Overall, the changes in spine density together with the reduction in mEPSC frequency, demonstrate a deficit in excitatory neurotransmission in the mPFC of GluN2C KO that occurs early during development.

### Altered excitatory and inhibitory synapses in the mPFC due to the loss of GluN2C subunits

**Figure 3:**
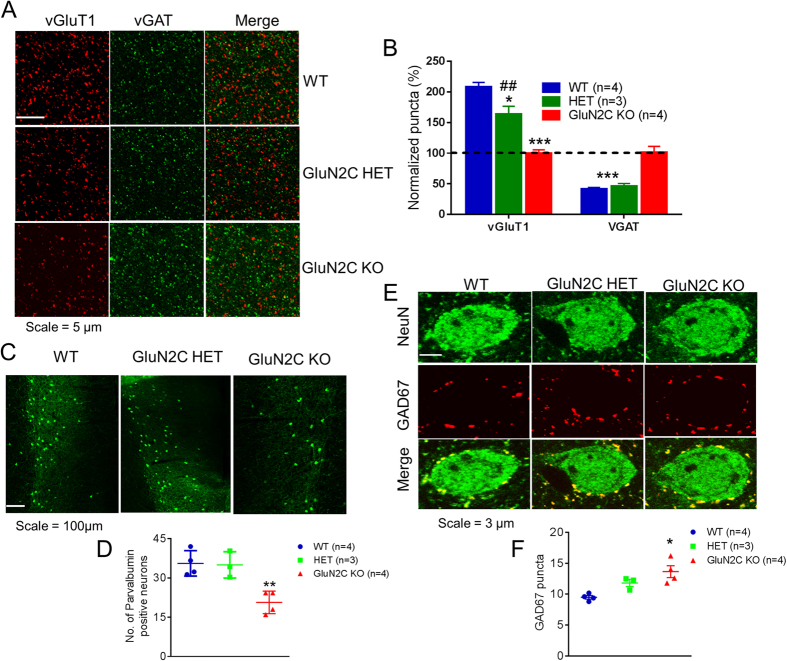
Excitatory-inhibitory synapses are altered in GluN2C knockout mice. (**A,B**) vGluT1 and vGAT puncta in layer V mPFC of WT, GluN2C HET and GluN2C KO mice. (**A**) Representative images of layer V mPFC at 10X (top) and 63X (bottom) indicating vGluT1 and vGAT puncta and (**B**) quantitative results. GluN2C HET and KO mice had a significantly lower number of vGluT1-positive puncta relative to WT controls (n = 3–4/genotype, one-way ANOVA followed by Bonferroni’s post-hoc test, *P < 0.05, ***P < 0.001). Knockouts also showed a significantly higher number of vGAT-positive puncta relative to WT controls (n = 3–4/genotype, Bonferroni’s post-hoc test, ***P < 0.001). (**C,D**) The number of PV-positive cells were significantly lower in the mPFC of GluN2C KO mice at P30 (Bonferroni’s post-hoc test, n = 3–4/genotype, **P < 0.01). (**C**) Representative images and (**D**) quantitative results. (**E**,**F**) The number of perisomatic GAD67 puncta on NeuN-labelled cells in layer V of the mPFC was significantly higher in GluN2C KO mice relative to WT controls (Bonferroni’s post-hoc test, n = 3–4/genotype, *P < 0.05). (**E**) Representative images at 63X indicating GAD67 puncta (red) on NeuN-positive cells (green) and (**F**) quantitative results.

In order to further establish changes in E/I balance in the mPFC we performed immunohistochemical analysis for markers of excitatory (vGluT1) and inhibitory (vGAT) synapses in this region. A significant genotype effect was observed in the analysis of vGluT1 puncta (Fig. 3A,B; one-way ANOVA, P < 0.0001, n = 4 for WT, 3 for HET and 4 for KO). We observed a significantly lower number of vGluT1-positive puncta in both GluN2C HET (Bonferroni post-hoc test, P < 0.05) and GluN2C KO mice (Bonferroni post-hoc test, P < 0.001), supporting our observation of an overall reduction in excitatory neurotransmission. A significant genotype effect was also observed in vGAT puncta (one-way ANOVA, P = 0.0005). GluN2C KO mice had a significantly higher number of vGAT-positive puncta compared to WT controls (Fig. 2E,F, Bonferroni post-hoc test, P < 0.001), consistent with an increase in mIPSCs in GluN2C KO. No change in vGAT puncta was observed in GluN2C HET in comparison to WT.

The activity of pyramidal neurons in the mPFC is modulated by the strong and synchronous inhibition by locally acting PV interneurons^29^. Interestingly, a decrease in the mRNA levels of GAD67 in PV-positive cells in the DLPFC^30^,^31^, as well as in PV mRNA itself^32^,^33^, has been reported in multiple cohorts of schizophrenic subjects. Moreover, alterations in E/I balance in the DLPFC have been proposed to emerge as a result of diminished activity of these interneurons^29^,^34^. Since the GluN2C subunit is expressed in PV interneurons in the mPFC^9^ and GluN2C mRNA expression was found to be lower in the DLPFC of human schizophrenic subjects^22^, it is likely that ablation of GluN2C could result in abnormalities in PV interneurons in this region. To test this hypothesis, we performed immunohistochemistry and analyzed the number of PV-labeled cells in the mPFC of WT, GluN2C HET and GluN2C KO mice. A significant genotype effect was observed in the number of PV-labeled neurons (Fig. 3A,B; one-way ANOVA, P = 0.0036, n = 4 for WT, 3 for HET and 4 for KO). We found a significant reduction in the number of PV-labeled cells in the mPFC of GluN2C KO mice at P30 as compared to WT controls (Bonferroni post-hoc test, P < 0.01). Additional immunohistochemical analysis at P30 revealed that GluN2C KOs have a significantly greater number of GAD67 puncta on pyramidal neurons in the mPFC compared to WT controls (Fig. 3E,F, P < 0.05, Bonferroni post-hoc test). Since in this region, PV interneurons have the predominant perisomatic inhibitory input onto pyramidal neurons, these data suggest potentially higher PV interneuron mediated inhibition in the mPFC of GluN2C KO mice. We also measured whether there were changes in somatostatin, a marker for somatostatin interneurons which are also abundant in mPFC. No change in number of somatostatin-positive neurons was observed in the mPFC of GluN2C KO (Supplementary Figure 3).

**Figure 4:**
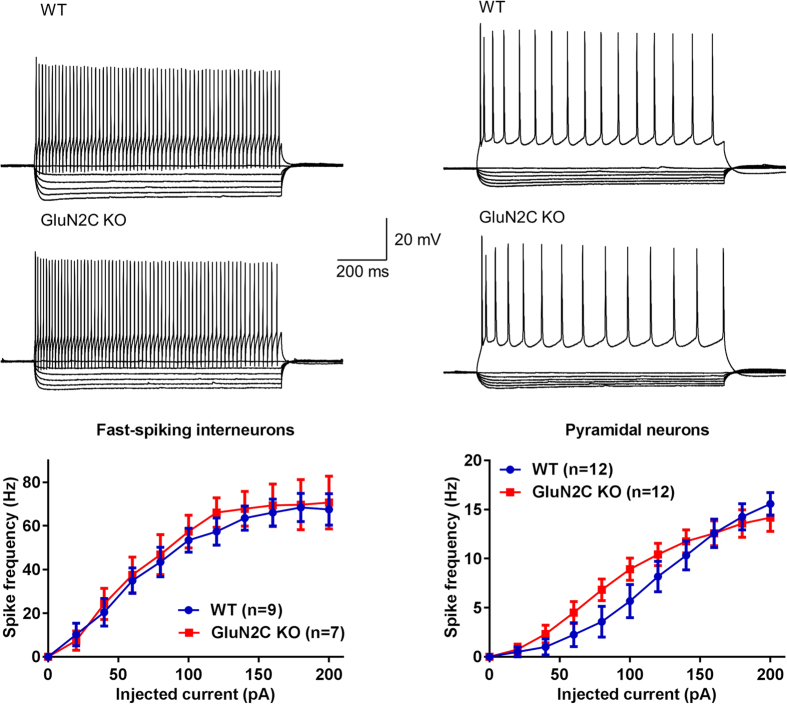
Deletion of GluN2C subunit does not affect excitability of fast-spiking interneurons or pyramidal neurons in medial prefrontal cortex. Whole-cell current clamp recording was performed from fast-spiking neurons and pyramidal neurons in layer V of medial prefrontal cortex. Current injection from −100 pA to 200 pA. No change in spike frequency was observed in either of the cell types due to deletion of GluN2C subunit. Other intrinsic properties and action potential characteristics were also unchanged (Table 1).

We next tested whether there are changes in excitability of fast-spiking interneurons and pyramidal neurons due to loss of GluN2C subunit. For these experiments current-clamp recordings were obtained from fast-spiking interneurons and pyramidal neurons in the layer V of mPFC. No change in the spike frequency was observed in GluN2C KO (Fig. 4). In addition, no change in other intrinsic characteristics including resting membrane potential and input resistance, as well as action potential characteristics was observed (Table 1). These findings together with the analysis of miniature and spontaneous currents indicate that homeostatic changes may maintain the excitatory-inhibitory balance at a circuit level despite changes in the total number of excitatory and inhibitory synapses.

### GluN2C subunit is critical to normal cortical neuronal oscillations

**Figure 5:**
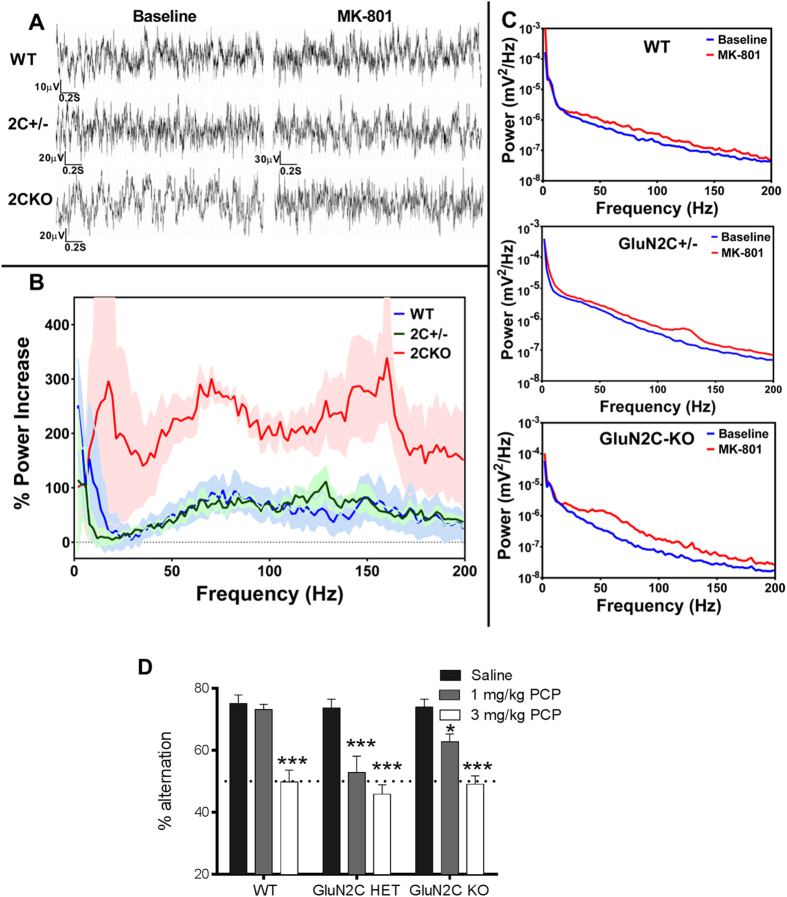
Abnormal neuronal oscillations and sensitivity to NMDAR channel blocker due to loss of GluN2C subunit. (**A**) Representative ECoG recordings of WT, GluN2C HET, and GluN2C KO mice display typical awake responses. (**B**) Representative power spectrum analyses of responses before and after MK-801 administration in WT, GluN2C HET, and GluN2C KO mice as indicated. (**C**) Average percent increase in the power spectrum in WT (n = 6), GluN2C HET (n = 4), and GluN2C KO mice (n = 4) in response to MK-801 administration (0.2 mg/kg IP). Shaded areas represent S.E.M. (**D**) Effect of phencyclidine on spontaneous alternation in Y-maze (n = 11–13 for WT, 11 for GluN2C HET and 12–13 for GluN2C KO). PCP (1 mg/kg) reduced the percent alternation in GluN2C HET and KO mice (Bonferroni’s multiple comparison, *P < 0.05, **P < 0.01) but not in GluN2C WT mice. At 3 mg/kg PCP induced impairment in spontaneous alternation in all genotypes (***P < 0.001).

Due to their unique biophysical properties such as reduced voltage-dependency due to a lower sensitivity to Mg^2+^-blockade as well as lack of desensitization^17^, GluN2C–containing receptors may be active without the requirement for prior depolarization and via spillover glutamate thereby leading to tonic activity, which can impact neuronal oscillations. In addition, synchronized activity of PV interneurons, wherein abnormalities were observed in GluN2C KO, is crucial to generation of gamma oscillations. A role for NMDAR in neuronal oscillations has been well-documented by administration of NMDAR antagonists which cause a significant increase in the power of oscillations, particularly in the gamma frequency band. Thus, as a measure of NMDAR-modulated oscillations, we evaluated NMDAR blockade-induced changes in neuronal oscillations in WT and GluN2C KO mice. Prior to MK-801 (0.2 mg/kg IP) administration, baseline oscillatory power appeared 140% greater in the GluN2C KO mouse than in the WT mouse in the 10–60 Hz frequency range and very similar at higher frequencies. Two-way ANOVA indicated that this was not significant; there was no interaction between frequency and genotype. However, when power was averaged in 10 Hz bands (0–10 Hz, 10–20 Hz, etc.), t-test with correction for multiple comparisons indicated that there was a significant increase in oscillatory power in the KO mouse for the 20–30 Hz band (P = 0.022), 30–40 Hz band (P = 0.028), and almost in the 40–50 Hz band (P = 0.051). We found that 30 minutes after MK-801 administration, there was a distinct increase in oscillatory power in both WT and KO mice (Fig. 5A). Two-way ANOVA did not show an interaction between genotype and frequency, however, one-way ANOVA analysis of power in the gamma band (30–100 Hz) shows that MK-801 increased gamma oscillatory power significantly more in GluN2C KO mice (224 ± 32%) than in WT (62 ± 25%; P = 0.0017) and in GluN2C HET mice (56 ± 10%; P = 0.0026). It is possible that the increase in neuronal oscillations in the GluN2C KO following MK-801 administration is due to the increased inhibitory tone in the GluN2C KO and the consequently greater disinhibition of excitatory neurons and increase in oscillations following the blockade of the remaining populations of NMDARs in interneurons of either cortex or thalamus.

As a separate test of the hypothesis that GluN2C KO mimics impairments seen in schizophrenia, we evaluated behavioral changes associated with schizophrenia. Specifically, we tested whether ablation of GluN2C would influence basal or NMDAR blocker (phencyclidine)-induced spontaneous alternation in the Y-maze, a test for working memory although it can be influenced by other factors such as repetitive behavior or hyperlocomotion. A significant genotype as well as genotype × drug effect was observed (two-way ANOVA; genotype × drug [F(4, 98) = 2.52, P = 0.0456], drug [F(2, 98) = 51.63, P < 0.0001], and genotype [F(2, 98) = 5.28, P = 0.0066]) (Fig. 5F, n = 11–13 for WT, 11 for GluN2C HET and 12–13 for GluN2C KO/treatment). Bonferroni’s multiple comparison revealed that while WT treated with 1 mg/kg PCP behaved similar to saline control, GluN2C HET and KO injected with 1 mg/kg PCP exhibit a significant deficit in spontaneous alternation compared to saline controls (P < 0.001 and P < 0.05 respectively). At the dose of 3 mg/kg PCP induced impairment in spontaneous alternation across all genotypes compared to respective saline controls (P < 0.001). These results indicate that GluN2C HET and KO mice exhibit higher sensitivity to NMDAR-channel blocker-induced deficits in spontaneous alternation.

### GluN2C HET and KO mice exhibit deficits in prepulse inhibition

**Figure 6:**
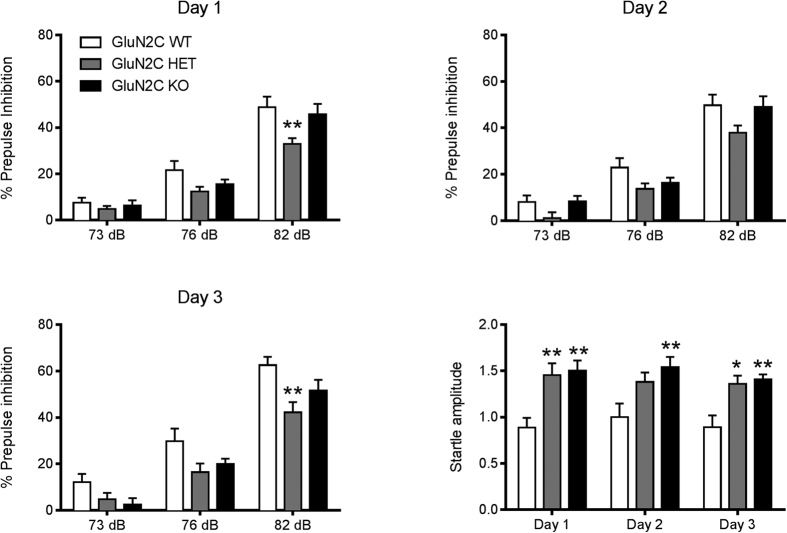
Disruption in PPI and startle responses in GluN2C HET and KO mice. PPI and startle responses of the 3 genotypes were recorded on 3 days of testing. No significant difference between the PPI response of WT and GluN2C KO mice was observed at either of the 3 decibel levels, whereas a significant reduction in PPI at 82 dB was observed for the HET group in comparison to WT controls (Bonferroni’s post-hoc test, **P < 0.01, n = 11 for WT, 13 for GluN2C HET and 12 for GluN2C KO). A significant increase in startle response was observed for HET and KO groups compared to WT controls (Bonferroni’s post-hoc test *P < 0.05, **P < 0.01).

We further evaluated whether there are changes in other cognitive behaviors due to ablation of GluN2C subunit. PPI of the startle reflex is a measure of sensorimotor gating, a phenomenon that underlies the ability to process and filter incoming information. Impairment in the PPI response is a characteristic behavior observed in schizophrenia patients^35^,^36^,^37^ as well as in NMDAR hypofunction models of SZ^38^. We therefore evaluated changes in startle response and PPI in GluN2C KO mice. The test for PPI was carried out on 3 consecutive days (n = 11 for WT, 13 for GluN2C HET and 12 for GluN2C KO; Fig. 6). Two-way repeated measures ANOVA with Dunnett’s multiple comparisons test revealed a significant decibel effect on all 3 days (F (2, 66) = 204.2–286.6, P < 0.0001). A significant genotype effect was observed on day 1 (F (2, 33) = 4.31, P = 0.0218), day 2 (F (2, 33) = 3.64, P = 0.0370) and on day 3 (F (2, 33) = 4.73, P = 0.0156), suggesting that the genotype influences PPI response. Bonferroni’s post-hoc test revealed a significant reduction in PPI response in the HET group compared to WT controls at 82 dB on day 1 (P < 0.01) and on day 3 (P < 0.01). On all 3 test days, the KO group showed a slight reduction in PPI at certain decibel levels, but none were found to be significant.

We also compared startle responses across the 3 genotypes on all 3 test days. Two-way repeated measures ANOVA revealed a significant genotype effect (F (2, 33) = 8.55, P = 0.0010) on startle amplitude. Bonferroni’s post-hoc analysis revealed that both HET and KO mice had a significantly higher startle amplitude compared to WT controls on all 3 days of testing except HET on day 2 (P < 0.05–0.01). Overall, our observations suggest that partial or complete deletion of GluN2C causes a modest reduction in PPI, and significantly increases the startle response. We also found that in a set of animals which were housed individually at the time of weaning, leading to social isolation, produced hyperactivity and social deficits in GluN2C KO mice but not in other genotypes (data not shown).

## Discussion

The reverberating circuits in the mPFC maintained by the interplay between excitatory and inhibitory neurotransmission regulate several behavioral phenotypes, including working memory and social behavior, which are relevant to neurodevelopmental disorders such as schizophrenia^39^. We found that ablation of the GluN2C subunit leads to remarkable abnormalities in the mPFC. In GluN2C KO mice, the decrease in frequency of mEPSCs in layer V pyramidal cells in the mPFC suggests lower excitatory network activity; this finding is also supported by a lower number of vGluT1-positive puncta and reduced dendritic spine density in layer III and V pyramidal cells, both of which reflect the number of glutamatergic synapses to pyramidal cells in this region. A trend toward lower basal dendritic spine density in layer III of the mPFC of GluN2C KO mice appears at P10, and progressively reduces to significance at P20 and P30. Incidentally, the onset of spine deficits in GluN2C KO also coincides with the adolescent onset of GluN2C expression in mice brain^10^ suggesting a critical role of GluN2C function in development during a period that coincides with a period of vulnerability in schizophrenia.

These results are partially, but not fully, consistent with a schizophrenia-like phenotype in the GluN2C KO mouse. We find that there is a reduction in PV-cell labelling consistent with schizophrenia and animal models of schizophrenia. In contrast, in the mPFC of the GluN2C KO mouse, we found a higher inhibitory tone and a greater number of inhibitory puncta on pyramidal neurons. A simple explanation for this finding is that a reduction in PV labeling does not necessarily result in lower inhibitory drive. Indeed, PV levels are known to change in an activity-dependent manner^40^, and lower PV levels suggest reduced firing of these interneurons. Thus one possibility is that the increase in number of inhibitory synapses may be a compensatory mechanism for lower firing of PV interneurons. Along those lines, deletion of PV has been shown to facilitate repetitive IPSCs with repeated stimulation, particularly at gamma frequencies^41^. Alternatively, the increase in frequency of mIPSCs in layer V pyramidal cells that we observe may be due to a higher inhibitory drive by interneurons other than those positive for PV, such as cholecystokinin, that are known to also target cell soma and proximal dendrites of these pyramidal cells, and that modulate the activity of PV cells in this region^29^. Although this hypothesis would explain the altered E/I balance as well as the reduction in PV labeling, further studies would be required to assess the involvement of GluN2C in different interneuron populations.

Neuronal oscillations are critical for cognition and working memory. Thus, changes in NMDAR-modulated neuronal oscillations would have important implications for behavior and cognition. Blockade of NMDAR activity *in vivo* causes a well-documented increase in oscillations, especially in the gamma frequency band^42^. This has been proposed to represent the blockade of NMDAR on PV-interneurons causing a reduction in inhibitory activity upon excitatory neurons, thus disinhibition. In the GluN2C KO mouse, NMDAR blockade causes enhanced gamma oscillations relative to those in WT mice. Thus, augmentation of NMDAR antagonist-induced oscillations following GluN2C ablation indicates that GluN2C containing receptors are not responsible for NMDAR antagonist induced oscillations in the GluN2C KO condition. Augmented oscillations might be explained by the increase in inhibitory tone in the GluN2C KO. In this condition, blockade of the remaining NMDAR in PV interneurons may possibly cause a greater disinhibition than seen in WT mice and hence a greater augmentation of gamma oscillations.

One of the limitations of our study is that the constitutive knockout nature of the model does not allow us to isolate the cell-type or circuit specific role of GluN2C subunit in the emergence of the cortical deficits. However, the GluN2C subunit is expressed rather sparsely and uniquely in the forebrain which allows us to make certain predictions. GluN2C subunit mRNA is reported in the PV interneurons in the cortex^9^ and NMDAR in PV interneurons are known to be critical to cortical function^11^,^12^,^13^. The reduction in PV labeling in the GluN2C KO as well as abnormal inhibitory tone suggest that GluN2C expression in PV interneurons may underlie the observed deficits in GluN2C KO. There are also alternative sites where GluN2C is enriched which can lead to cortical deficits. Specifically, GluN2C subunit is enriched in the relay cells of the MDT that project onto neurons in the mPFC, as well as the nRT, that modulates the activity of the MDT^8^,^43^,^44^. Thus abnormal MDT input to the pyramidal neurons in the mPFC may result in the lower spine density observed in GluN2C KO. Moreover, the MDT neurons also have inputs on PV interneurons^45^. Additional studies are required to precisely address whether GluN2C expression in thalamus controls cortical function and cognition.

Our previous and current results demonstrate that mice deficient in GluN2C subunit exhibit impairments in working memory, associative learning, as well as sensorimotor gating that are relevant to the cognitive deficits in neurodevelopmental disorders such as schizophrenia^7^; (Fig. 5). In addition, the hyper-responsiveness to social isolation- and PCP-induced social and cognitive deficits and alterations in basal neuronal oscillations and MK-801-induced augmentation of neuronal oscillations is also relevant to schizophrenia. Overall, GluN2C deficient mice have face validity for schizophrenia and it is conceivable that a reduction in GluN2C expression reported in schizophrenia patient brains^18^,^19^,^20^,^21^,^22^ may partly underlie the symptoms in schizophrenia. In relation to this conclusion it should be noted that the E/I imbalance towards greater inhibition is contrary to the current understanding of schizophrenia. One possibility is that the greater inhibition represents an early pathology and/or homeostatic mechanism to normalize cortical circuitry and requires further evaluation. Other groups have previously proposed a potential role of GluN2C/GluN2D subunits in schizophrenia^15^,^46^,^47^. This notion emanates from the unique expression pattern of these subunits in interneuron subpopulations and in relevant thalamic nuclei^8^,^9^,^44^. Overall, our current findings together with other recent observations with GluN2D KO mice^48^ present strong evidence that these subunits are indeed critical to emergence of schizophrenia-like phenotypes.

## Materials and Methods

### Animal husbandry

We used the GluN2C knockout/*n*β-galactosidase knockin mouse^10^ which were backcrossed on to C57BL/6 background (95% and remaining 129 Sv/Ev). Experimental procedures were performed on male GluN2C wildtype (WT), heterozygous (HET), and knockout (KO) littermate mice. Mice (6–8 weeks old) were age matched (within one week of age) for different behavioral tests. Unless specified otherwise, animals were group-housed on a 12:12 light/dark cycle with *ad libitum* access to food and water. At least 3 or more litters formed the subjects of each of the experimental group and experiments were conducted in at least 2 or more batches and assimilated. Different groups of mice were used for different experiments. All procedures were approved by the Creighton University Institutional Animal Care and Use Committee and conformed to the NIH Guide for the Care and Use of Laboratory Animals.

### Drug information

Phencyclidine (PCP, P3029, Sigma-Aldrich, MO), (+)-MK-801 hydrogen maleate (M107, Sigma-Aldrich, MO) was dissolved in sterile isotonic saline and administered at a volume of 0.1 ml via intraperitoneal (IP) injection.

### Electrophysiology

Whole-cell electrophysiology was performed as previously described^7^ with minor modifications. Briefly, acute prefrontal cortex slices were obtained from mice at postnatal days (P) 22–28. After isoflurane anesthesia mice were decapitated and brains were removed rapidly and placed in ice-cold artificial cerebrospinal fluid (ACSF) of the following composition (in mM): 130 NaCl, 24 NaHCO_3_, 3.5 KCl, 1.25 NaH_2_PO_4_, 0.5 CaCl_2_, 3 MgCl_2_ and 10 glucose saturated with 95% O_2_/5% CO_2_. 300–350 μm thick coronal sections were collected using vibrating microtome (Leica VT1200, Buffalo Grove, IL, USA). Whole-cell patch recordings were obtained from layer V prelimbic cortical pyramidal neurons in voltage-clamp configuration with an Axopatch 200B (Molecular Devices, Sunnyvale, CA, USA). Glass pipettes with a resistance of 5–8 mΩ were filled with an internal solution consisting of (in mM) 110 cesium gluconate, 30 CsCl, 5 HEPES, 4 NaCl, 0.5 CaCl_2_, 2 MgCl_2_, 5 BAPTA, 2 Na_2_ATP, and 0.3 Na_2_GTP (pH 7.35). QX314 (5 mM) was added to block voltage-gated sodium channels. The recording ACSF contained (in mM) 1.5 CaCl_2_ and 1.5 MgCl_2_. The mEPSCs were recorded at −70 mV holding potential in the presence of 1 μM tetrodotoxin and 100 μM picrotoxin and mIPSCs were recorded at 0 mV holding potential in the presence of 1 μM tetrodotoxin, 20 μM NBQX and 100 μM AP5. Whole-cell recordings with a pipette access resistance less than 20 mΩ and that changed less than 20% during the duration of recording were included. Signal was filtered at 2 kHz and digitized at 10 kHz using an Axon Digidata 1440 A analog-to-digital board (Molecular Devices, CA). Recordings were analyzed using Minianalysis (Synaposoft, Atlanta, GA, USA) with an amplitude threshold set at 6 pA, and frequency, amplitude and decay of mEPSCs were determined. A total of 3–4 animals were used for each genotype for mEPSC and a similar number for mIPSC recordings, and a total of 11–20 neurons from each genotype were used for analysis.

For current-clamp recordings, electrodes were filled with (in mM) 105 K-gluconate, 30 KCl, 10 HEPES, 10 Na_2_-phosphocreatine, 4 Na_2_ATP, and 0.3 Na_2_GTP (pH 7.2). To characterize the membrane properties of neurons, hyper- and depolarizing current steps were applied for 1 second in 20-pA increments from −100 pA to 200 pA. Input resistance was measured from the slope of a linear regression fit to the voltage-current relation in a voltage range hyperpolarized of membrane potential. Spike properties were quantified using the first evoked spike. Spike amplitude and the afterhyperpolarization were measured relative to the spike threshold. Spike half width was measured at its half-amplitude. The adaptation ratio was calculated as the ratio between the first and the last interspike interval during a depolarizing current step 60 pA above the rheobase. Firing frequency was calculated in hertz as a ratio between number of spikes and current step duration.

### Diolistic labeling and dendritic spine analysis

Diolistic labeling for analysis of dendritic spines was performed as previously described^49^. Tungsten particles coated with DiI (Molecular Probes, Life Technologies, Grand Island, NY, USA) were delivered into fixed coronal sections (150 μm thickness) using a Helios Gene Gun system (Bio-Rad Laboratories, Hercules, California, USA) and allowed to diffuse for 24–48 hr at 4 °C, followed by fixing in 4% PFA for 1 hr. Imaging of labeled sections was performed using LSM 510 confocal microscope and 63 x oil objective (Zeiss, Chester, VA, USA). The dendrite that was clearly connected to the soma and fully separable from crossing dendrites was cropped. The cropped dendrite was scanned at 0.1 μm intervals along the z-axis (depending on the depth of the dendrite) using the same objective. The dendritic spines of the 1^st^ branch point of first order dendrites were used for the analysis in layer V of mPFC. The filament module of Imaris software 7.6.3, 3-D imaging software (Bitplane, South Windsor, CT, USA) and Imaris XT were used to quantify the spine density and morphology and for spine classification. Rules for spines classification are previously described^49^. For each neuron, 2–4 dendrites were analyzed. For each animal at least 15–20 neurons were analyzed, and a total of 3–4 animals were used for each group.

### Golgi staining and dendritic spine analysis

Golgi staining for visualization of dendritic spines was performed as previously described^49^, as per the manufacturer’s protocol. Brains from WT and GluN2C KO mice were collected and placed in 5 ml of impregnation solution (1:1 mixture of Solution A + Solution B) for 14 days. Brains were then transferred to Solution C for 72 hours, and frozen for ~1 minute in isopentane at −70 °C. Frozen brains were sectioned coronally (100–150 μm) using a cryostat (Leica CM 1900, IL). Sections were collected on gelatin-coated glass slides and stained, dehydrated in graded concentrations of ethanol (50%, 75%, 95%) and cleared with xylene. For analysis of dendritic spines, sections were imaged on Axioscope Z 1051–078 microscope (Zeiss, Chester, VA, USA) with 100X oil immersion objective using Q-Capture PRO7 software. Intact pyramidal neurons in layer II–III of the medial prefrontal cortex were chosen for analysis. Multiple parameters were compared between WT and GluN2C KO mice. For basal dendrites, 1^st^ order branches ~40 μm away from the cell soma were selected for analysis. For oblique apical dendrites, 1st order branches on the apical dendrite at least 50 μm away from the cell soma were chosen for analysis. A total of 10 neurons per parameter for each animal were analyzed, and 3–4 animals were used for each group.

### Immunohistochemistry

Immunohistochemistry was performed as previously described^7^,^49^. Mice were transcardially perfused with 4% PFA in 0.1 M phosphate buffer (PB) and brain collected and stored overnight in the same fixative at 4 °C. Brains were then transferred successively into solutions of 10%, 20% and 30% sucrose in 0.1 M PB before freezing in isopentane at −30 °C to −40 °C. For immunohistochemistry 20 μm thick coronal sections were cut using a cryostat (Leica CM 1900). After washing thrice for 5 mins each with 0.01 M PBS, sections were incubated in blocking solution containing 10% normal goat serum (NGS) (for vGluT, NeuN, GAD67), or 10% normal donkey serum (NDS) (for vGAT, PV, somatostatin) (or both for double labeling) in 0.25% Triton-X in 0.01 M PBS (PBST) for 1 hr at room temperature. Following blocking, sections were incubated overnight at 4 °C in primary antibodies at appropriate concentrations in PBST (mouse anti-GAD67, 1:500, Millipore; rabbit anti-parvalbumin, 1:5000, Swant; rabbit anti-somatostatin14, 1:1000, Peninsula Laboratories International; mouse anti-NeuN labeled with Alexa Flour 488, 1:500, Chemicon International; mouse anti-vGluT1, 1:500, Millipore; rabbit anti-vGAT, 1:500, Millipore). The following day, sections were washed 6 times for 5 mins each in 0.01 M PBS, and incubated with the appropriate secondary antibodies conjugated to AlexaFluor 488 (donkey anti-rabbit IgG-conjugated Invitrogen, CA, 1:500 in PBST), or AlexaFluor 647 (goat anti-rabbit IgG-conjugated, Invitrogen, CA, 1:500 in PBST) for 2 hours at room temperature in dark. Sections were then washed thrice for 5 mins each in 0.01 M PBS, mounted on pre-cleaned glass slides and coverslipped with Fluoromount-G (SouthernBiotech, AL). Images were captured using a confocal microscope (Leica FV1000). Images from a single confocal plane were captured with a 63X oil immersion objective (NA 1.4). A stack of three images beginning at the surface of the neuron was captured by z-axis scanning at 1 μm intervals for each neuron. All images were acquired using the same parameters. GAD67, vGAT and vGluT1 puncta (<2 μm away from the soma membrane; 10–80 pixels) were counted using NIH ImageJ software. The image capture and analysis were done by investigators blind to the genotype.

### Electrocorticography (ECoG)

Mice were surgically-implanted with tripolar electrodes (MS333/2, Plastics One, Roanoke, VA, USA) under xylazine/ketamine/acepromazine anesthesia as required by IACUC regulations. Two holes were made in the skull 3 mm posterior to bregma at 1 mm and 2.5 mm lateral. Two electrodes were placed in the medial hole onto the dura surface near the retrosplenial cortex and the third electrode was placed in the lateral hole for ground. The electrodes were secured to the skull with dental cement. After 7 days of recovery, ECoG recordings were made with a DP-311 differential amplifier (Warner Instruments) with high pass/low pass filters set at 0.1 and 300 Hz and digitized/recorded (Digidata 1400, pClamp 10, Molecular Devices). Following 30 minutes of baseline recordings, animals were injected i.p. with MK-801 or saline and recorded at 40 to 60 minutes post-injection corresponding to maximal drug response. Power spectrum analysis was performed with Clampfit (Molecular Devices) using a Hamming window with 50% overlap.

### Behavioral analysis

#### PCP-induced deficits in spontaneous alternation

WT, GluN2C HET and GluN2C KO mice were injected with saline or PCP (1 or 3 mg/kg) and 10 min later placed in the Y-maze. Spontaneous alternations were recorded and analyzed as described previously^50^.

#### Prepulse-inhibition and startle response

The prepulse inhibition test was performed using six Startle Monitor Systems (Kinder Scientific, Julian, CA). Each unit was housed in a sound attenuation cabinet (35.56 cm wide × 27.62 cm deep × 49.53 cm high). A speaker mounted on the cabinet’s ceiling was used to generate acoustic stimuli (70 dB–120 dB white noise). The startle response was measured by a piezoelectric sensing platform on the floor, which was calibrated daily. During testing, mice were placed in a rectangular box made of transparent Plexiglas (19 cm wide × 9.8 cm deep × 14.6 high) with an adjustable ceiling positioned atop the box, providing only limited restraint while prohibiting ambulation. Animals were first handled individually for two days, for approximately 2 min each day to minimize stress during behavioral testing, and testing was carried out for 3 consecutive days post handling. The PPI session, conducted on each of the 3 test days, lasted approximately 18 min. Each session began with a 5 minute period of 70 dB background noise (which continued throughout the duration of the session) followed by four different trial types- pulse alone trials and three types of prepulse+pulse trials, which consisted of a 20 ms 73, 76, or 82 dB prepulse (3, 6, and 12 dB above background) followed 100 ms later by a 120 dB pulse. Each session was divided into 4 blocks. Blocks 1 and 4 were identical, each consisting of 4 pulse alone trials. Blocks 2 and 3 were also identical and each consisted of 8 pulse alone trials and 5 of each prepulse+pulse trial type. A total of 54 trials were presented during each test session. Trials within each block were presented in a pseudorandom order and were separated by a variable inter-trial interval averaging 15 s (ranging from 9 to 21 s). Startle magnitude was defined as the maximum force (measured in Newtons) applied by the animal to the startle apparatus recorded over a period of 100 ms beginning at the onset of the pulse stimulus. Startle responses from testing blocks 2 and 3 were used to calculate percent prepulse inhibition (%PPI) for each acoustic prepulse trial type:

% PPI = ((mean startle response to 120 dB pulse alone-mean startle response following a prepulse)/mean startle response to 120 dB pulse alone) * 100.

### Statistical analyses

Data were analyzed using unpaired t-test with Welch’s correction or one-way or two-way ANOVA with Bonferroni or Dunnett’s *post-hoc* test. For histochemical analysis multiple values obtained from randomly obtained images for one animal were averaged to generate a single value per mouse. For PPI data repeated measures analysis of variance with genotype as a between-subjects factor and prepulse intensity as a within-subject factor was used to determine main effects. *Post-hoc* individual comparisons were carried out to evaluate genotype effect at each decibel. Data was analyzed using Prism 4 (GraphPad Software Inc., San Diego, CA, USA).

## Additional Information

**How to cite this article**: Gupta, S. C. *et al*. The NMDA receptor GluN2C subunit controls cortical excitatory-inhibitory balance, neuronal oscillations and cognitive function. *Sci. Rep.* **6**, 38321; doi: 10.1038/srep38321 (2016).

**Publisher's note:** Springer Nature remains neutral with regard to jurisdictional claims in published maps and institutional affiliations.

## Supplementary Material

Supplementary Dataset

Supplementary Information

Supplementary Data File

## Figures and Tables

**Table 1:** Electrophysiological characteristics of layer V fast-spiking interneurons and pyramidal neurons in mPFC.

	Fast-spiking interneurons		Pyramidal neurons	
	WT (n = 9)	GluN2C KO (n = 7)	WT (n = 12)	GluN2C KO (n = 12)
Resting membrane potential (mV)	−58.5 ± 1.2	−57 ± 0.9	−63.1 ± 2	−60.7 ± 1.5
Input resistance (MΩ)	330 ± 22	330 ± 38	172 ± 15	221 ± 22
Spike threshold (mV)	−34.8 ± 0.8	−35.8 ± 0.9	−36 ± 1.5	−34.6 ± 1.2
Spike amplitude (mV)	77.4 ± 3.2	80.8 ± 3.2	105 ± 2.1	103 ± 1.8
Spike half width (ms)	0.86 ± 0.04	0.81 ± 0.07	1.42 ± 0.04	1.62 ± 0.06
After hyperpolarization (mV)	38.6 ± 1.4	40.2 ± 1.4	10.1 ± 0.9	11.3 ± 1.1
Adaptation ratio	0.6 ± 0.04	0.55 ± 0.04	0.32 ± 0.04	0.37 ± 0.04
