# Supplementary material for: The NMDA receptor GluN2C subunit controls cortical excitatory-inhibitory balance, neuronal oscillations and cognitive function
Source: Sci Rep. 2016 Dec 6;6:38321. doi: 10.1038/srep38321 (PMC5138829; doi:10.1038/srep38321)

The NMDA receptor GluN2C subunit controls cortical excitatory-inhibitory balance, neuronal oscillations and cognitive function

Subhash C. Gupta[1], Aparna Ravikrishnan[1], Jinxu Liu[1], Zhihao Mao[2], Ratnamala Pavuluri[1], Brandon G. Hillman[1], Pauravi Gandhi[1], Dustin J. Stairs[3], Ming Li[4], Rajesh R. Ugale[5], Daniel T. Monaghan[2] and Shashank M. Dravid[1*]

Author affiliations:

[1]Department of Pharmacology, [3]Psychology, Creighton University, Omaha, NE 68178 USA, [2]Department of Pharmacology and Experimental Therapeutics, University of Nebraska-Medical Center, Omaha, NE, [4]Department of Psychology, University of Nebraska-Lincoln, Lincoln, NE 68588 USA, [5]Department of Pharmaceutical Sciences, R.T.M. Nagpur University, Nagpur, Maharashtra 440033 India.

Running title: GluN2C subunit regulates cortical E/I balance

Manuscript: Figures: 6; Tables: 1

Word count: Abstract: 242, Introduction: 497, Discussion: 1193.

Corresponding Author: Dr. Shashank M. Dravid, Department of Pharmacology, Creighton University, School of Medicine, 2500 California Plaza, Omaha, NE 68178, Phone: 402-280-1885, Fax: 402-280-2142, Email: ShashankDravid@creighton.edu

Supplementary figure 1. (A) No significant differences in the frequency, amplitude or decay of sEPSCs of layer V pyramidal neurons in the mPFC were observed between WT and GluN2C KO mice (n=8 cells from 3-4 mice/genotype). Representative traces **(a)**, cumulative probability plots for inter-event interval and amplitude and histograms for frequency and amplitude in inset **(b, c)** and histograms for decay **(d)**. **(B)** No significant differences in the frequency or decay and a modest reduction in amplitude of sIPSCs of layer V pyramidal neurons in the mPFC in GluN2C KO mice (n=8 cells from 3-4 mice/genotype). Representative traces **(a)**, Cumulative fraction plots for inter-event intervals and amplitude and histograms for frequency and amplitude in the inset **(b, c)** and decay kinetics **(c, f)**. Only amplitude was modestly but significantly lower in GluN2C KO sIPSC (unpaired t-test with Welch's correction, *P = 0.0110).

Supplementary figure 2: Changes in dendritic spine density in different layers, and for different spine types in the mPFC of GluN2C KO mice. (A) Dendritic spine density was significantly reduced in layer V of the mPFC in GluN2C KO mice; this reduction was observed for all spine types (*P = 0.01295, stubby and P = 0.0101 thin and long spines and ***P = 0.0007 for filopodia-like spines, unpaired t-test with Welch's correction, n = 4/genotype), except for mushroom spines. **(B, C)** No significant difference in the oblique apical dendritic spine density was observed between WT and GluN2C KO mice at P10, P20 or P30. **(B)** Representative images of Golgi-stained sections and **(C)** quantitative results.

Supplementary figure 3: No change in somatostatin labeling in GluN2C KO. The number of somatostatin-positive cells in the mPFC of GluN2C KO was not different from wildtype.

Supplementary figure 1

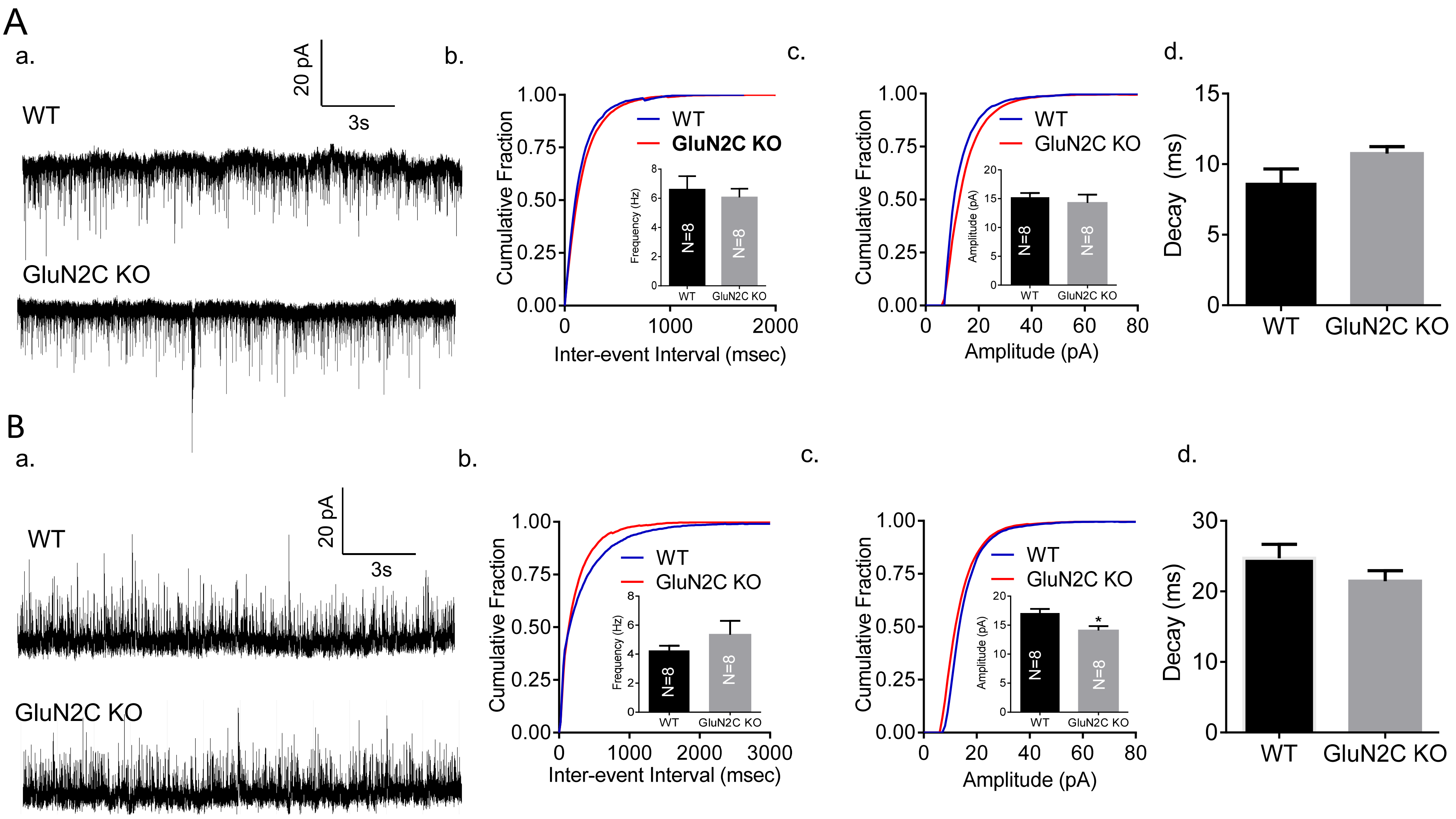

Supplementary figure 2

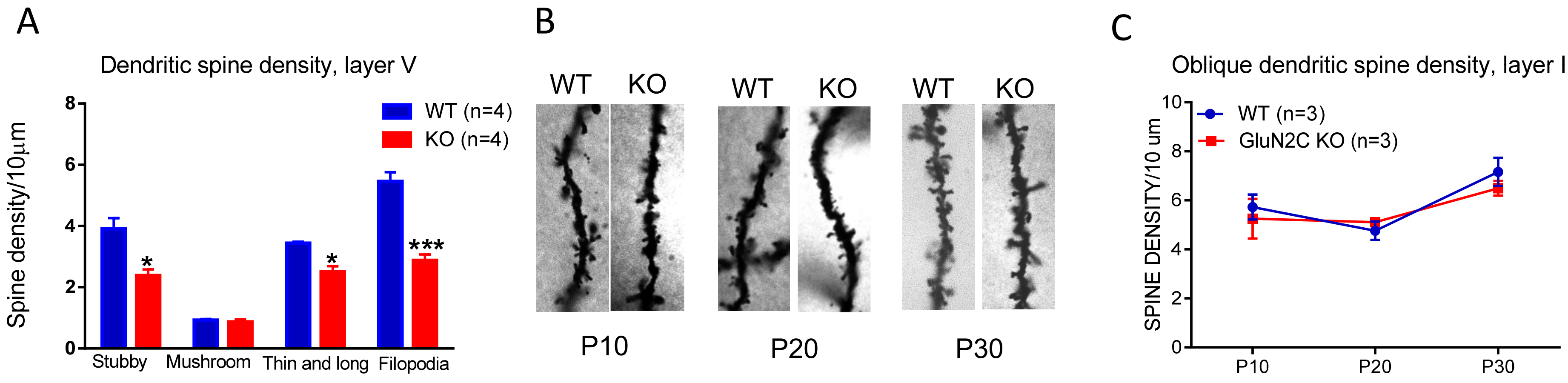

Supplementary figure 3

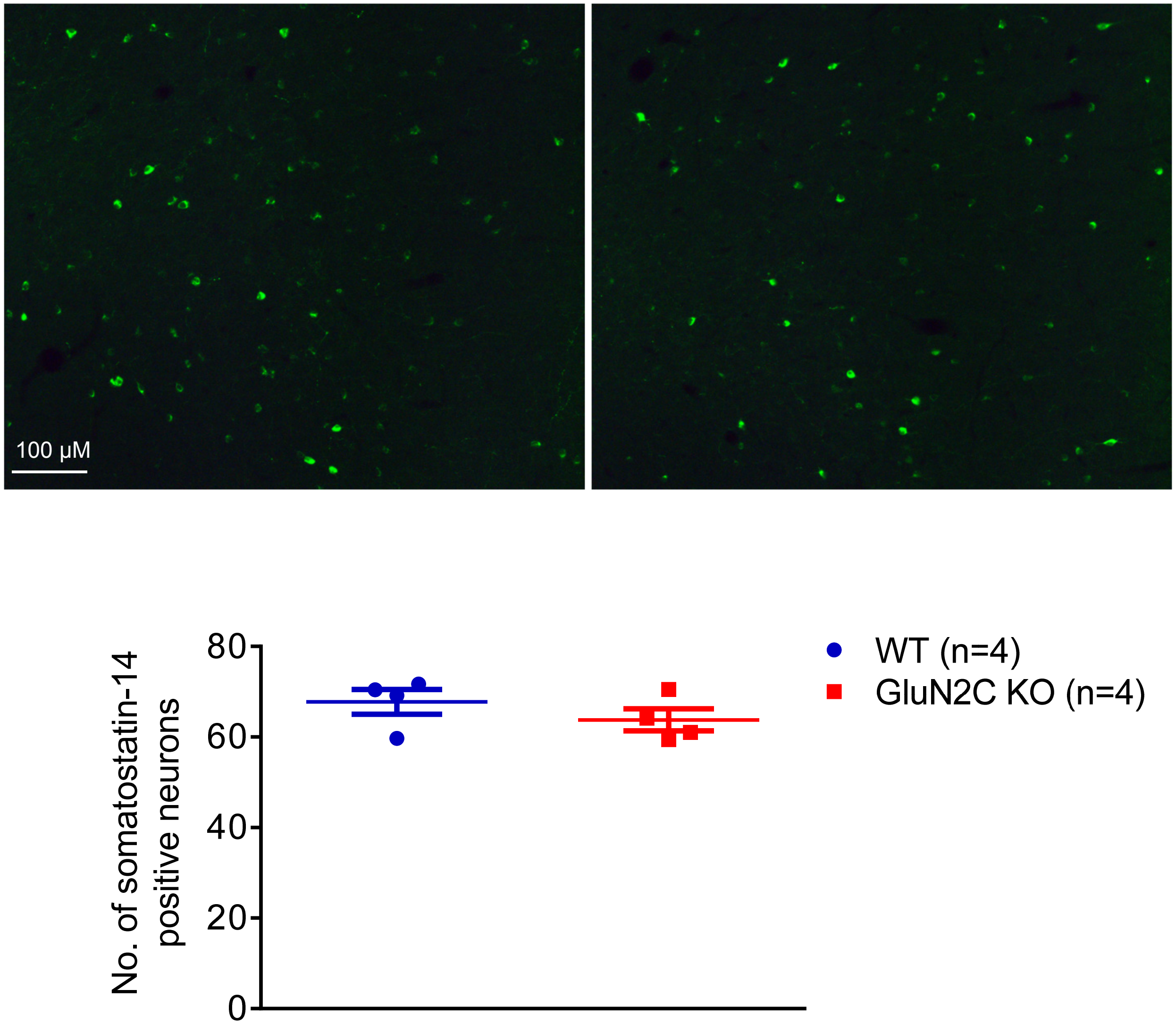

Figure 2A

Dendritic spine data in mPFC of wild type mice

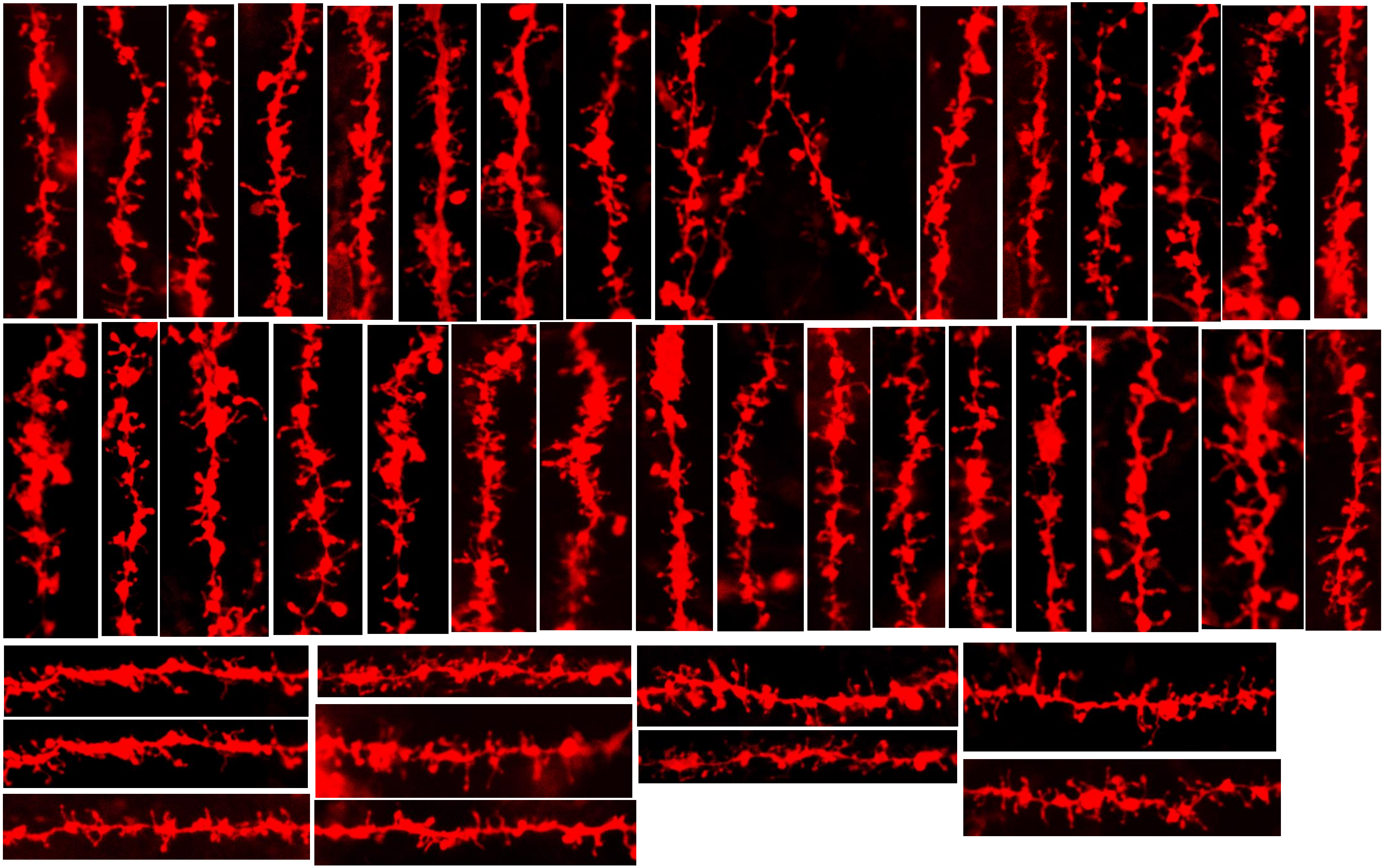

Figure 2A

Dendritic spine in GluN2C KO mice

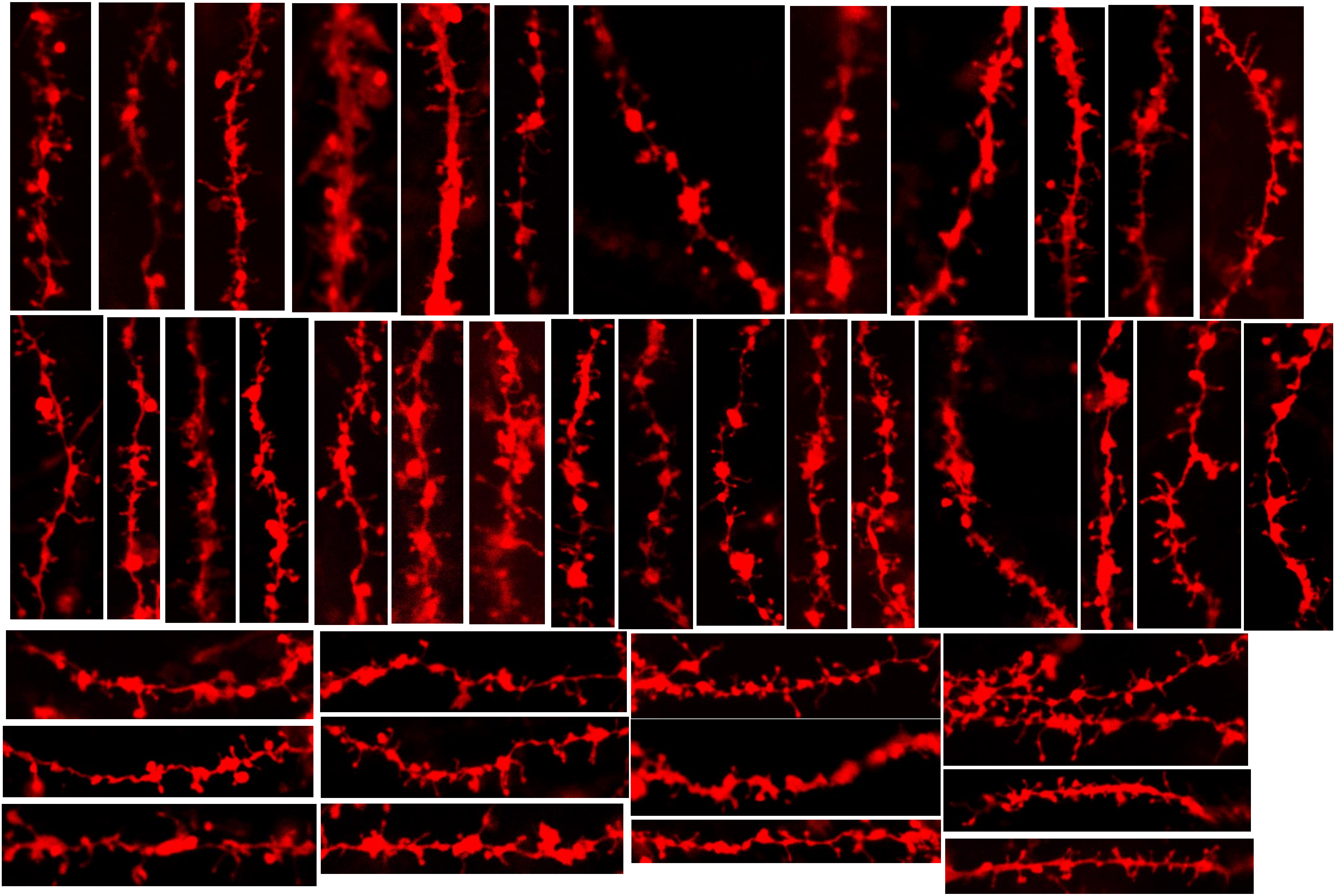

Figure 3A

WT (vGluT1-VGAT)

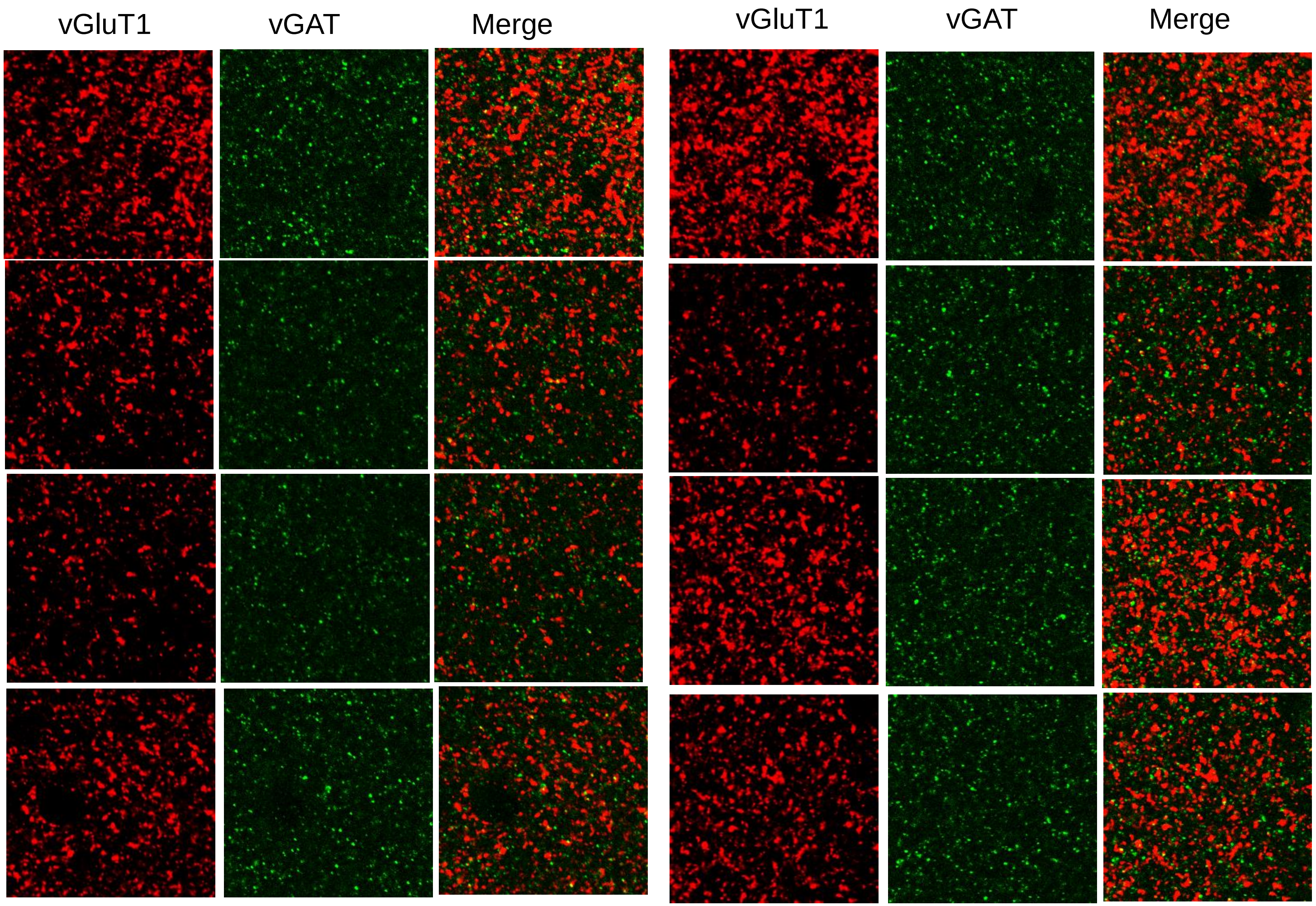

Figure 3A

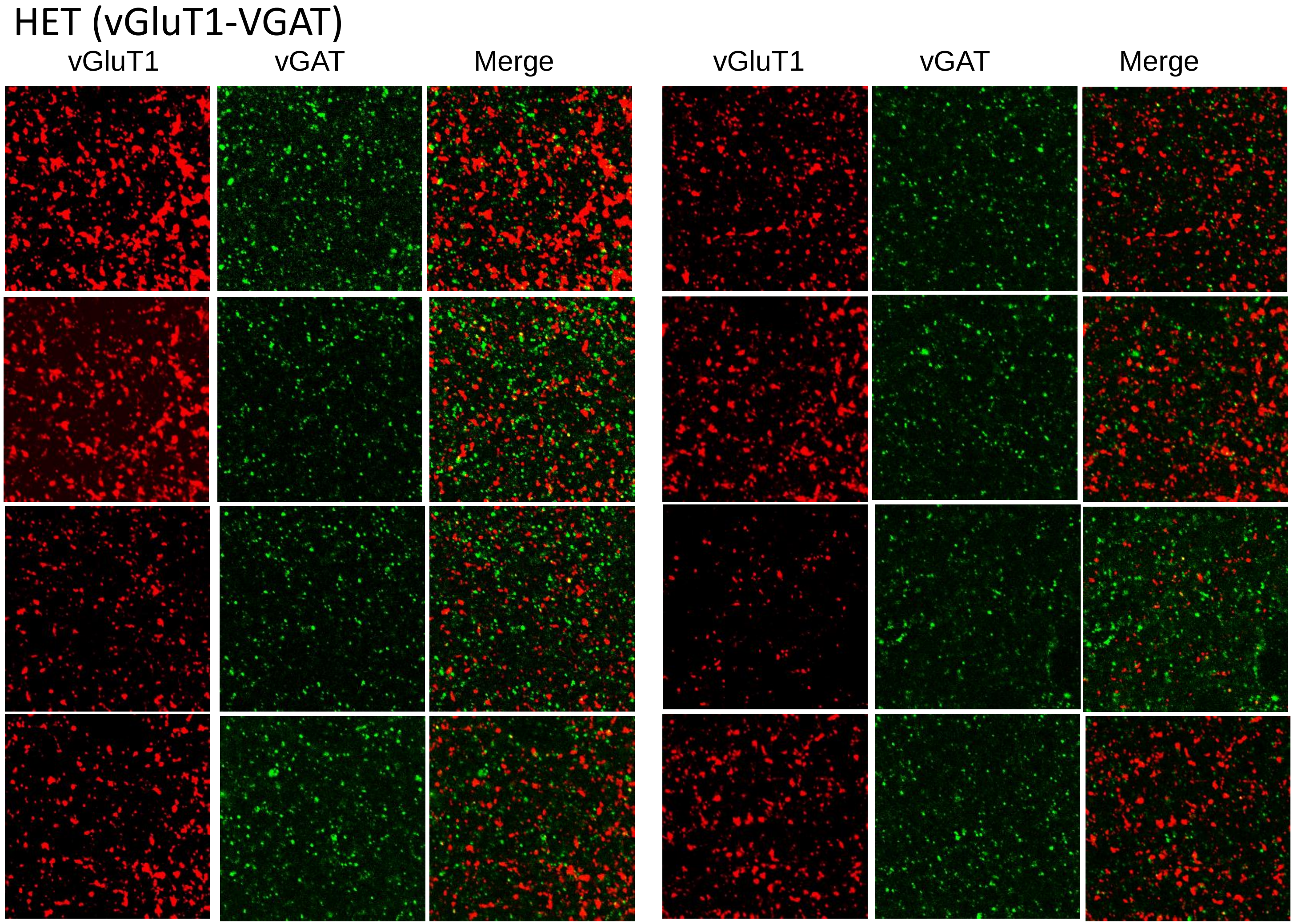

Figure 3A

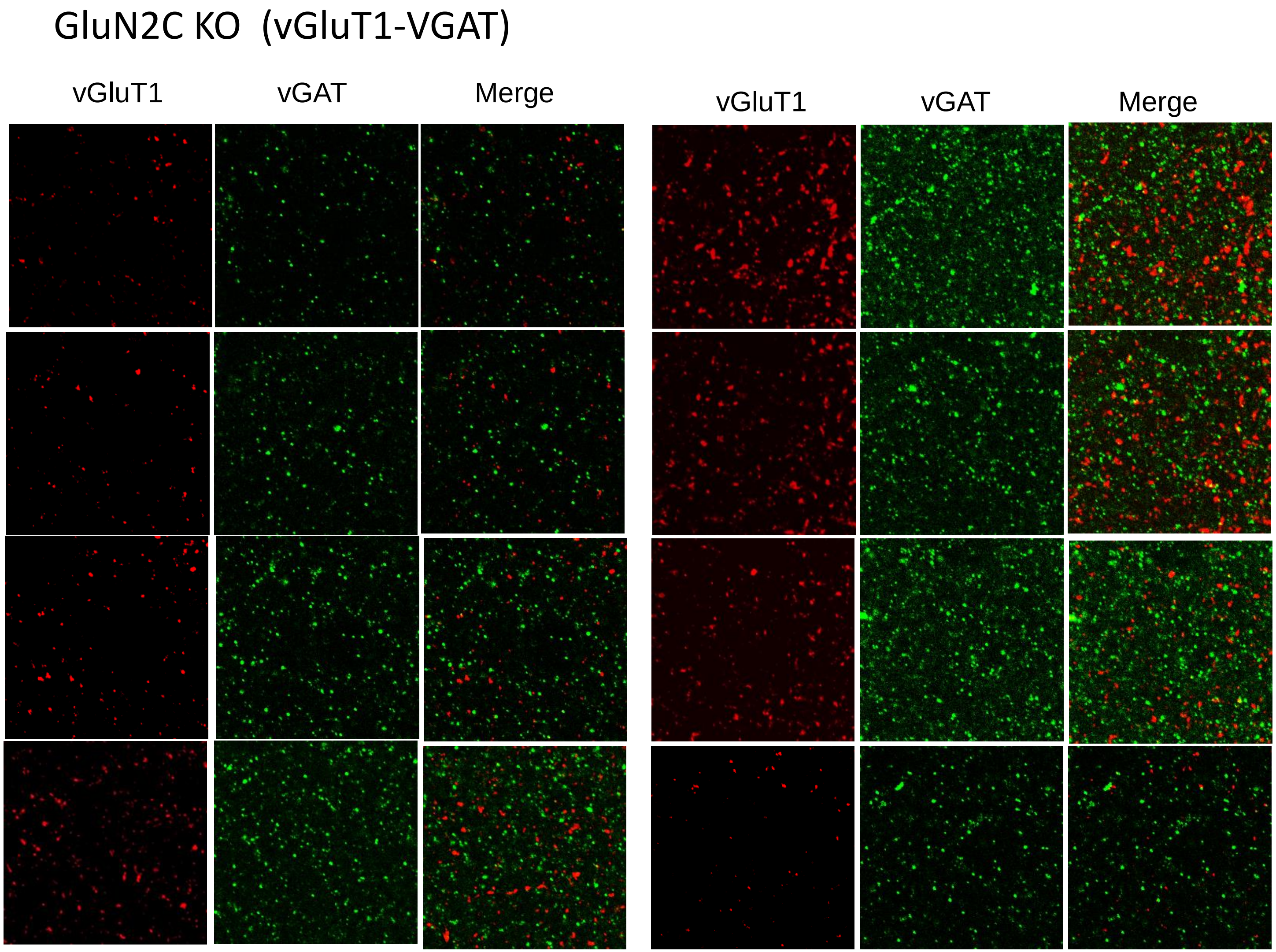

Figure 3C

PV-WT

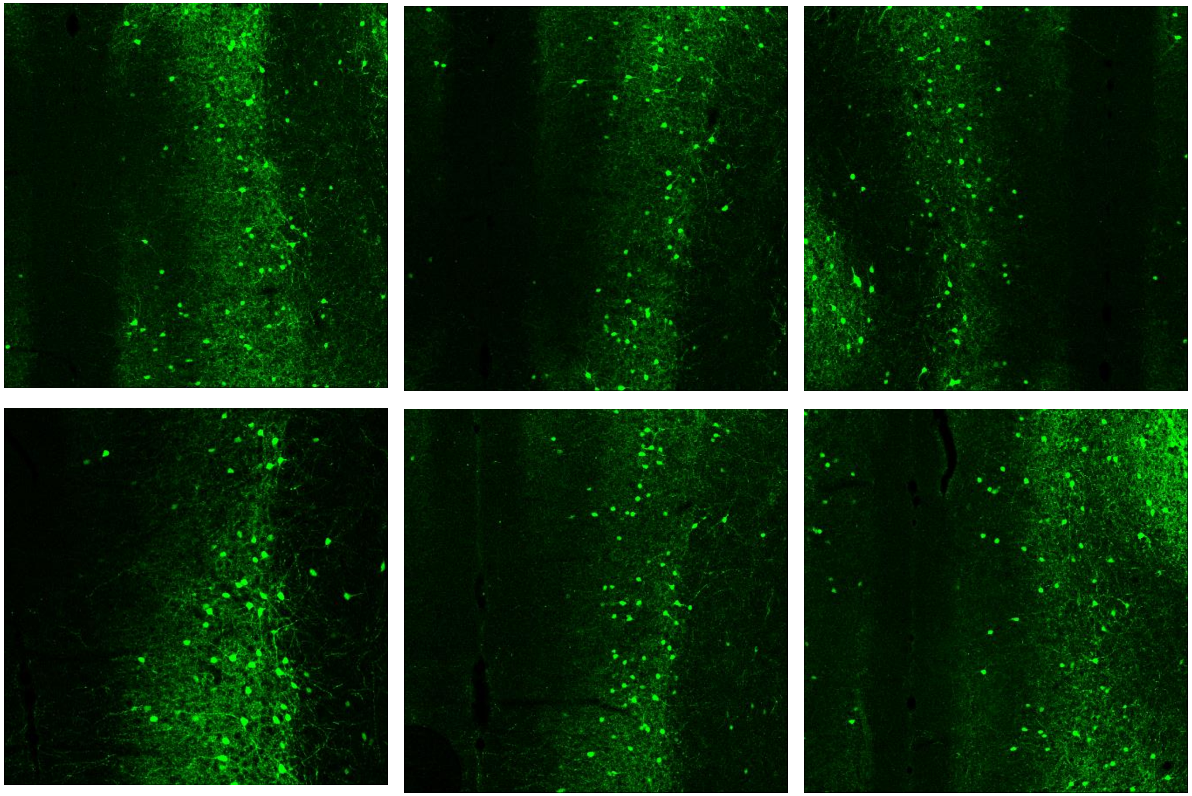

Figure 3C

PV-HET

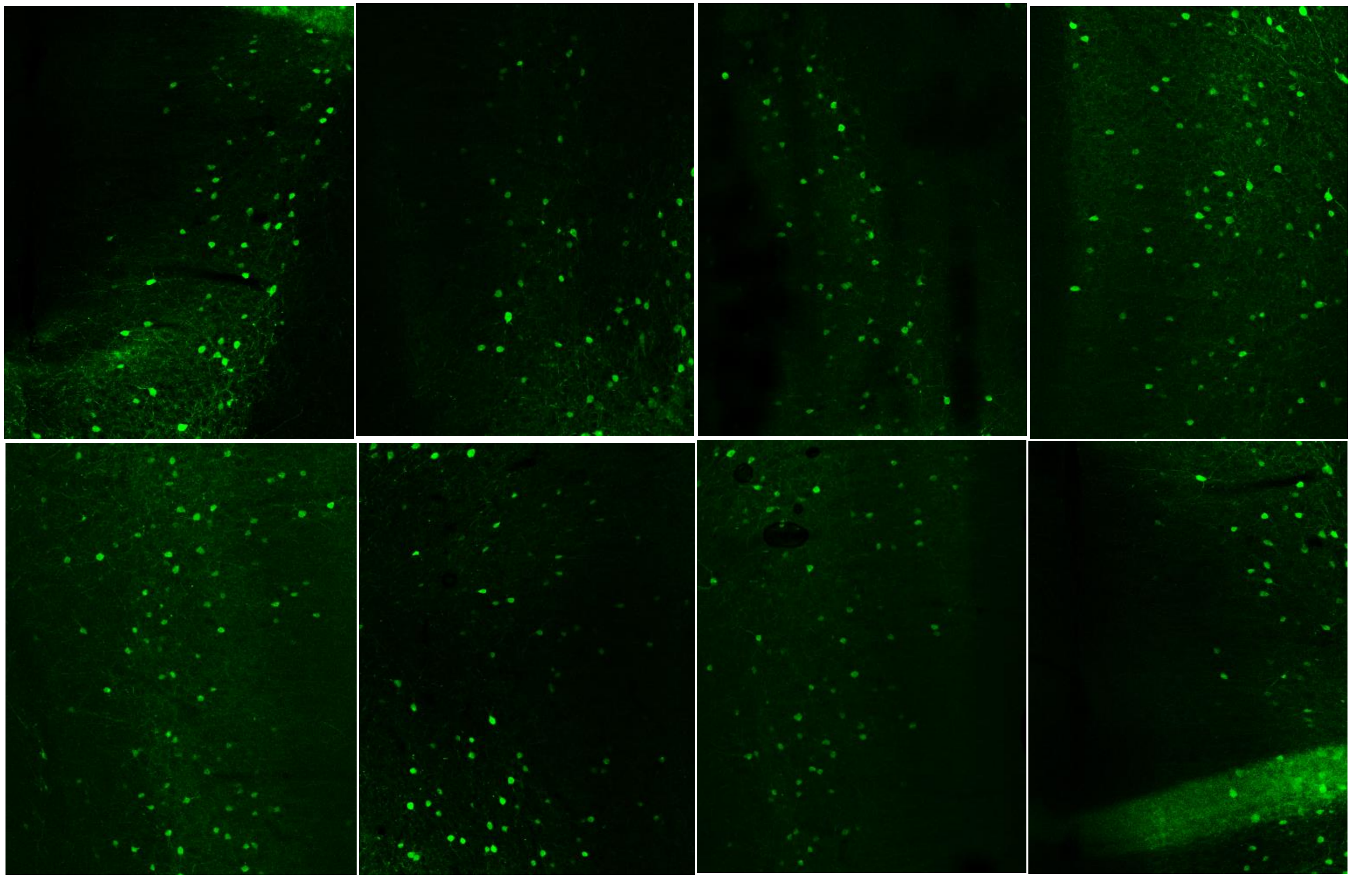

Figure 3C

PV-GluN2C KO

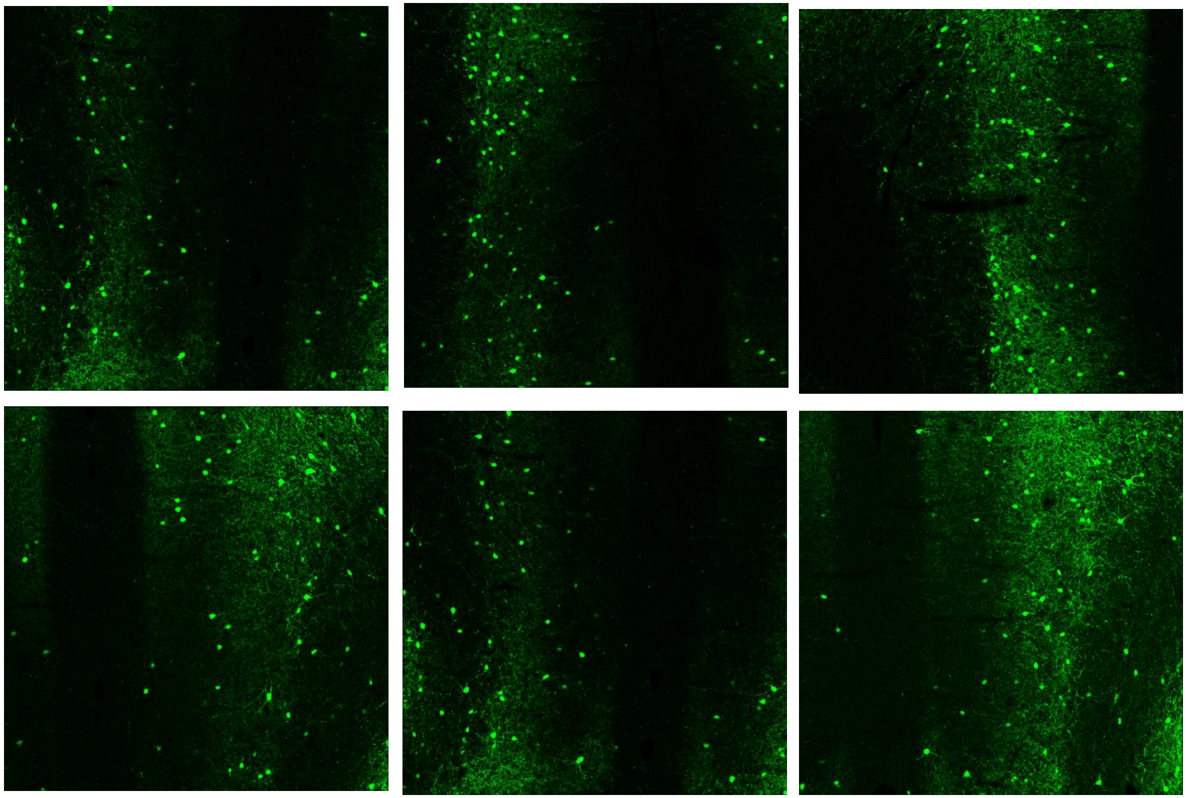

Figure 3E

WT-NeuN and GA67 staining

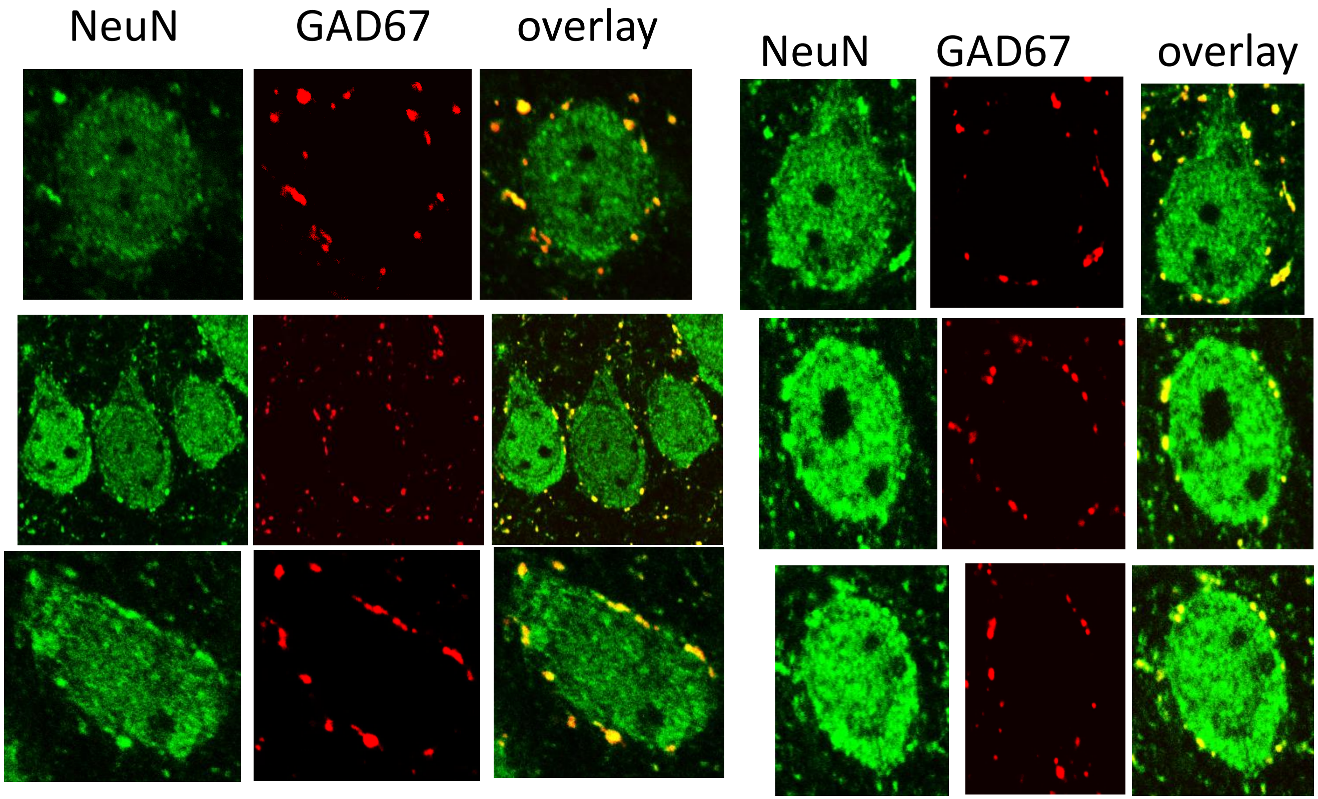

Figure 3E

GluN2C HET-NeuN and GA67 staining

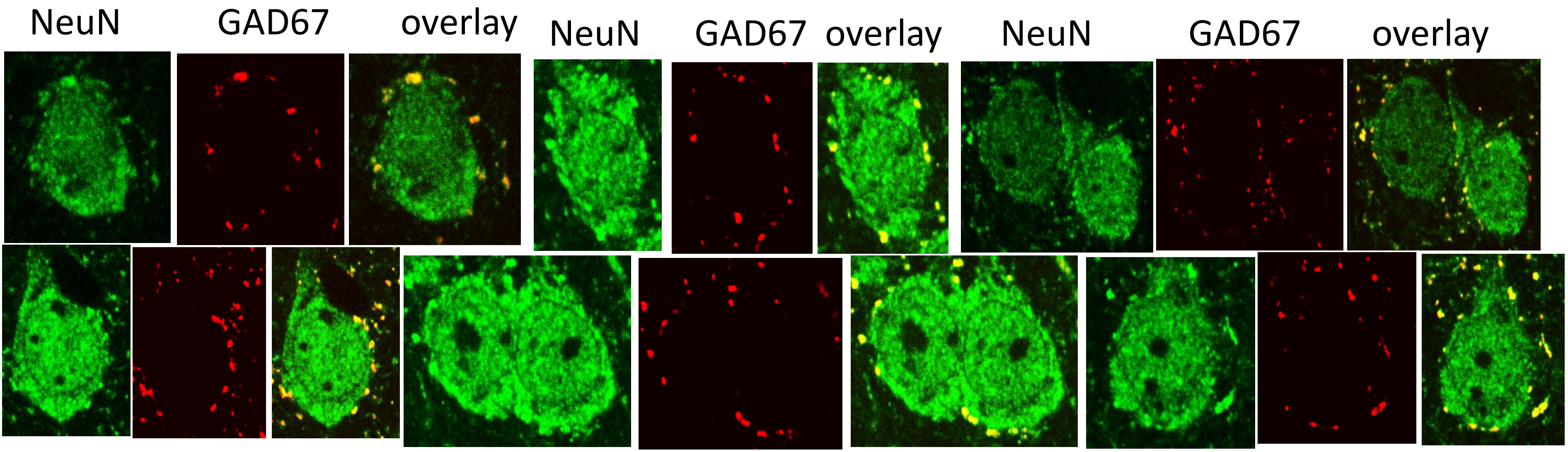

Figure 3E

GluN2C KO- NeuN and GA67 staining

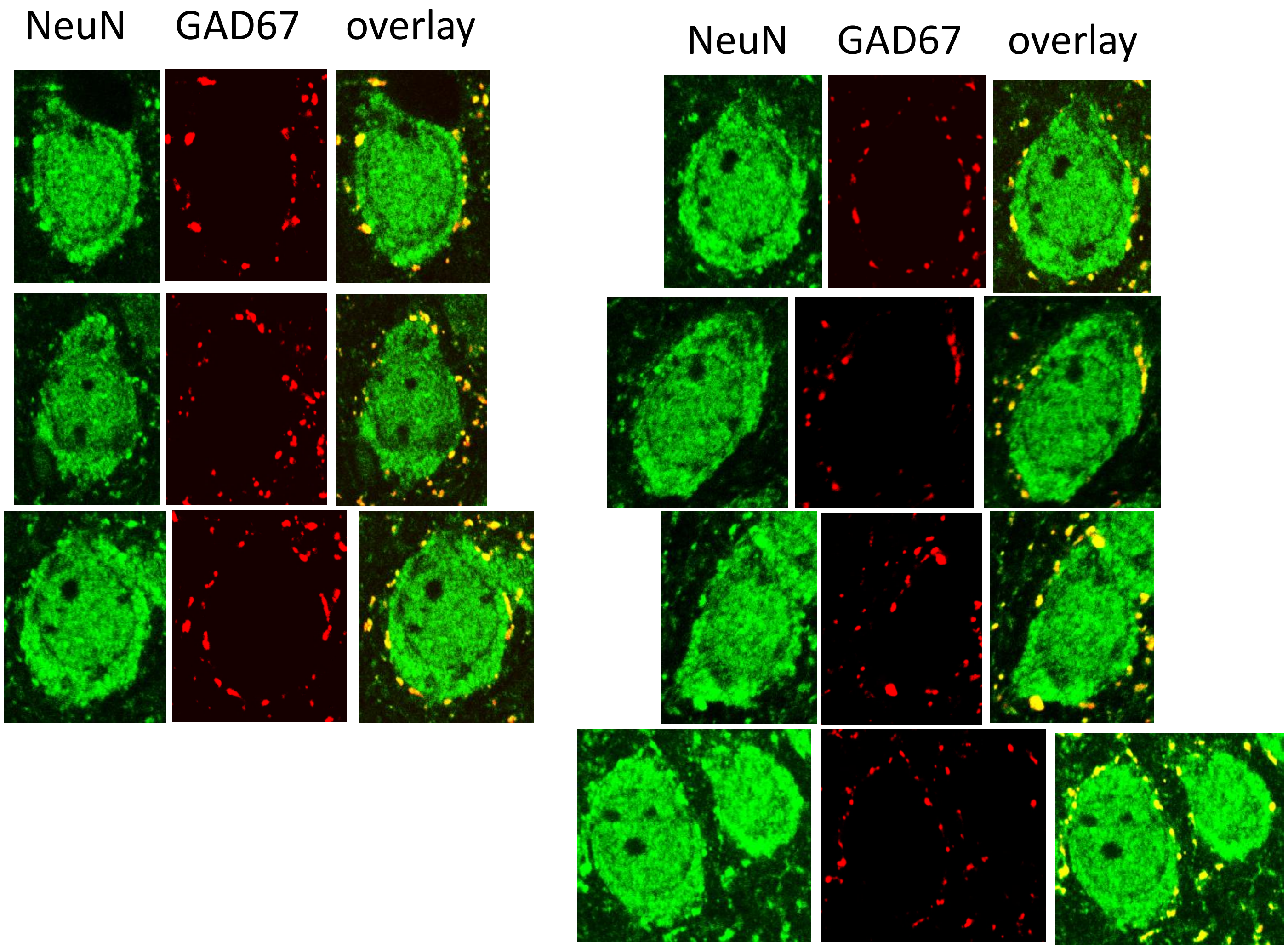

Supplement: Supplementary Dataset [file srep38321-s1.pdf]
